# Zoantharian Endosymbiont Community Dynamics During a Stress Event

**DOI:** 10.3389/fmicb.2021.674026

**Published:** 2021-05-28

**Authors:** Yu Fujiwara, Iori Kawamura, James Davis Reimer, John Everett Parkinson

**Affiliations:** ^1^Molecular Invertebrate Systematics and Ecology Laboratory, Department of Chemistry, Biology, and Marine Science, Faculty of Science, University of the Ryukyus, Nishihara, Japan; ^2^Nakajima Suisan Co. Ltd., Tokyo, Japan; ^3^Tropical Biosphere Research Center, University of the Ryukyus, Nishihara, Japan; ^4^Department of Integrative Biology, University of South Florida, Tampa, FL, United States

**Keywords:** light intensity, quantitative PCR, reciprocal transplant, Symbiodiniaceae, *Zoanthus sansibaricus*

## Abstract

Coral reefs are complex ecosystems composed of many interacting species. One ecologically important group consists of zoantharians, which are closely related to reef-building corals. Like corals, zoantharians form mutualistic symbioses with dinoflagellate micro-algae (family Symbiodiniaceae), but their associations remain underexplored. To examine the degree to which zoantharians exhibit altered symbiont dynamics under changing environmental conditions, we reciprocally transplanted colonies of *Zoanthus sansibaricus* between intertidal (2 m) and subtidal (26 m) depths within a reef in Okinawa, Japan. At this location, *Z. sansibaricus* can associate with three Symbiodiniaceae species from two genera distributed along a light and depth gradient. We developed species-specific molecular assays and sampled colonies pre‐ and post-transplantation to analyze symbiont community diversity. Despite large environmental differences across depths, we detected few symbiont compositional changes resulting from transplantation stress. Colonies sourced from the intertidal zone associated with mixtures of a “shallow” *Symbiodinium* sp. and a “shallow” *Cladocopium* sp. independent of whether they were transplanted to shallow or deep waters. Colonies sourced from the subtidal zone were dominated by a “deep” *Cladocopium* sp. regardless of transplant depth. Subtidal colonies brought to shallow depths did not transition to the presumably high-light adapted shallow symbionts present in the new environment, but rather bleached and died. These patterns mirror observations of highly stable coral-algal associations subjected to depth transplantation. Our results indicate that *Zoanthus*-Symbiodiniaceae symbioses remain stable despite stress, suggesting these important reef community members have relatively low capacity to shuffle to more stress-tolerant micro-algae in response to ongoing climate change.

## Introduction

Climate change continues to threaten environmentally-sensitive marine mutualisms, the most ecologically important of which are associations between reef-building corals (order: Scleractinia) and dinoflagellate micro-algae (family: Symbiodiniaceae). These nutritional symbioses form the foundation of tropical reef ecosystems, creating reef habitat in nutrient-poor waters *via* light-enhanced calcification (Roth, 2014). Such partnerships are sensitive to biotic and abiotic stressors, which can drive symbiont loss – a process termed coral bleaching (Fitt et al., 2001). Although bleached corals may not lose all of their symbionts, they are nevertheless compromised, and often expire or suffer reproductive consequences if conditions do not improve and the symbiont community does not recover (Baird and Marshall, 2002). During stress events, partner fidelity can vary greatly across different coral holobionts (the host and its microbes). Some corals exhibit a “shuffling” response, whereby the numerically dominant micro-algal symbiont changes to favor a more resilient species (Bay et al., 2016). Other corals are more stable, such that the dominant symbiont never alters even during bleaching events (Thornhill et al., 2006). This range of dynamics has been recorded across many different scleractinian-Symbiodiniaceae partnerships under both natural and experimental stress conditions.

However, scleractinian corals are not the only organisms on reefs that associate with Symbiodiniaceae, nor are they the only organisms that can bleach. Other sessile cnidarian hosts include octocorals (soft corals), as well as the hexacorallian sea anemones (order Actiniaria), and zoantharians (Trench, 1974). Zooxanthellate zoantharians in the genera *Zoanthus* and *Palythoa* are often common components of coral reef ecosystems in both the Atlantic and Indo-Pacific oceans (Karlson, 1980; Sebens, 1982; Burnett et al., 1997; Irei et al., 2011), but have received comparatively little attention with respect to their symbiont dynamics (but see Reimer et al., 2007). One such symbiotic zoantharian is *Zoanthus sansibaricus*, a species that is widely distributed across coral reefs in the Indo-Pacific (Reimer et al., 2017b). It can be found in a variety of environments – from the shallow intertidal zone to mesophotic depths (>50 m) – and is considered to be a generalist species (Kamezaki et al., 2013; Albinsky et al., 2018).

At one reef site (Manza) on the west coast of the main island of Okinawa, Japan, *Z. sansibaricus* colonies are present on the reef flat in intertidal and shallow subtidal waters as well as on the outer reef wall up to depths > 35 m (Kamezaki et al., 2013). Morphological, phylogenetic, and population genetic data indicate that *Z. sansibaricus* at Manza exhibit no obvious differences across depths and therefore together represent a homogenous population of a single species (Reimer et al., 2004; Kamezaki et al., 2013; Wham et al., 2013; Wham, 2015; Albinsky et al., 2018). However, the host can associate with at least three Symbiodiniaceae lineages that likely represent distinct species (Wham et al., 2014; Wham, 2015). Two of the lineages are primarily found in the shallows (*Symbiodinium* A1z in direct sunlight and *Cladocopium* C1z-intertidal in shadow; Reimer, 2008), while the other is primarily found at greater depth (*Cladocopium* C1z-subtidal), suggesting that they are adapted to different light levels and possibly different temperatures (Kamezaki et al., 2013; Leal et al., 2016).

We have previously examined Symbiodiniaceae biogeography and bleaching patterns among zoantharians (Parkinson et al., 2016; Noda et al., 2017; Reimer et al., 2017a), but to our knowledge, no studies have attempted to track zoantharian symbiont community dynamics throughout a stress event. As a result, it remains unclear to what extent zoantharian-Symbiodiniaceae associations may shuffle or remain stable in response to stressors associated with climate change. To fill this gap in our understanding, we reciprocally transplanted colonies of *Z. sansibaricus* from shallow and deep locations at Manza, exposing them to novel light and temperature conditions. We tracked symbiont community composition – the proportion of each Symbiodiniaceae species in each colony – using species-specific molecular markers. We detected almost no shuffling 1 month after transplantation, with excessive mortality in the deep-to-shallow (DS) transplants, indicating that the association is relatively stable despite stress. Therefore, *Z. sansibaricus* holobionts are unlikely to acclimate to climate change *via* alterations in symbiont community composition.

## Materials and Methods

All raw data and the R code used to generate the results can be found in the Supplementary Figure S1 and online at https://github.com/parkinson-lab.

### Study System

The study site was located at Manza, Okinawa Island, Japan (26° 50'N, 127° 85'E; for map, see Figure 1 of Kamezaki et al., 2013). Manza features a reef lagoon with a gradual shallow slope leading to a precipitous drop-off starting at approximately 10 m depth. During a typical summer, the light and temperature levels in the shallow intertidal zone are greater and more variable than in the deep dropoff zone. To quantify these differences, *in situ* data loggers (Onset Computer Co., Massachusetts) were deployed from 19 August to 2 September, 2015, and environmental readings were recorded hourly. At Manza, *Z. sansibaricus* exhibits a discontinuous distribution, with an “intertidal” (<2 m) group and a “subtidal” (7–35+ m) group (for images, see Figure 2 of Kamezaki et al., 2013). Based on previous ITS2 genotyping, the numerically dominant symbiont within intertidal *Z. sansibaricus* colonies is typically *Symbiodinium* sp. (ITS2 type A1z; *sensu* Reimer, 2008) or a shallow *Cladocopium* sp. (ITS2 type C1z-intertidal; *sensu* Reimer, 2008; Kamezaki et al., 2013). The subtidal *Z. sansibaricus* colonies are usually dominated by a distinct deep *Cladocopium* sp. (ITS2 type C1z-subtidal; *sensu* Reimer, 2008; Kamezaki et al., 2013). As yet, none of these symbiont lineages have formal names. For clarity and pending taxonomic description, hereafter, we refer to the three primary Symbiodiniaceae species at this location as A1z-shallow, C1z-shallow, and C1z-deep.

**Figure 1:**
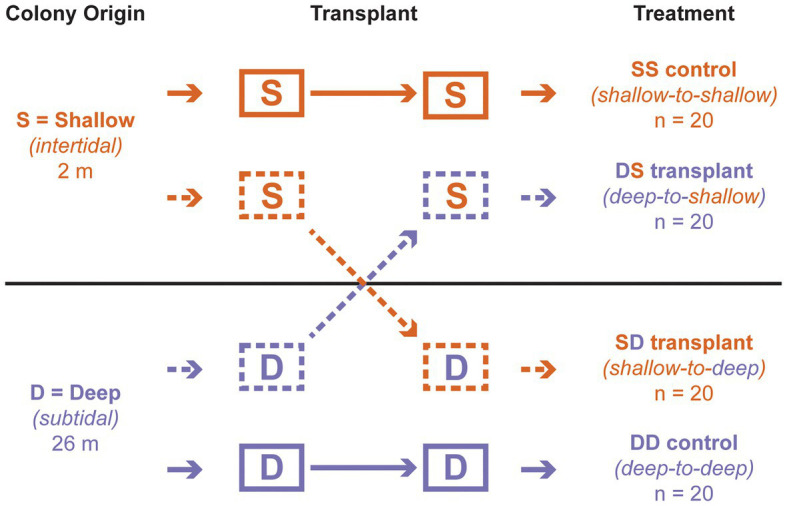
Reciprocal transplant design. The first letter of each treatment indicates depth of origin, while the second indicates depth of transplantation. “S” corresponds to “shallow” (intertidal; 2 m); “D” corresponds to “deep” (subtidal; 26 m).

**Figure 2:**
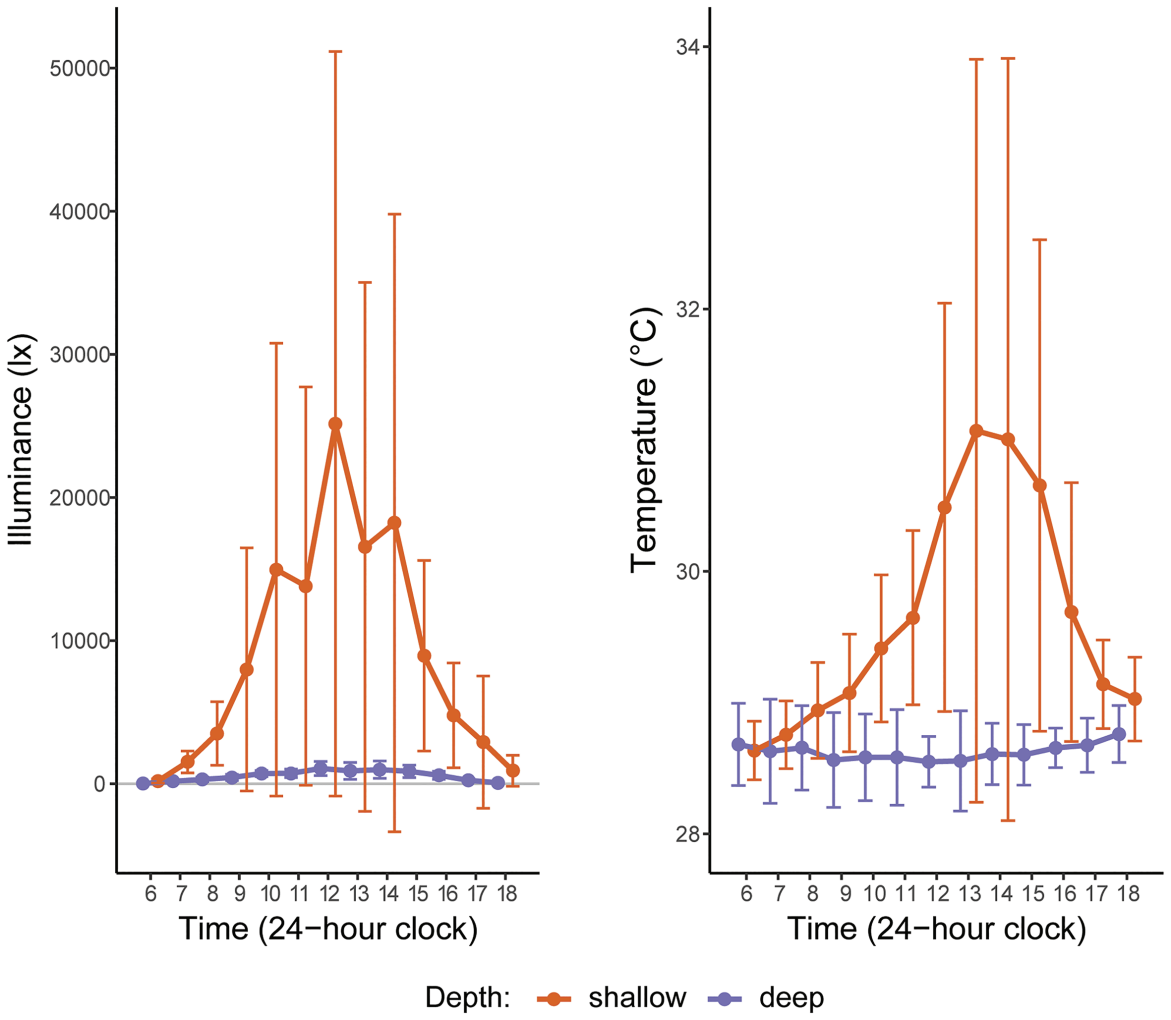
Hourly light (illuminance) and temperature readings at two depths in Manza, Okinawa Island, Japan, averaged over 2 weeks in Summer 2015. “Shallow” corresponds to the intertidal (2 m); “deep” corresponds to the subtidal (26 m). The gray horizontal line indicates zero illuminance. The large error bars for shallow depth reflect high hourly variation in these metrics, as expected in the intertidal zone.

### Reciprocal Transplants

Reciprocal transplant experiments were carried out from 24 July to 10 October, 2013. Substrates with whole *Z. sansibaricus* colonies (*n* = 40 per depth) were collected from the intertidal (2 m) or subtidal (26 m) using a hammer and chisel and brought to the surface. Pieces of substrate with colonies attached (~5 cm in diameter) were adhered to four concrete blocks (two per depth of origin) by applying Krazy Glue (Toagosei America, Inc., North Carolina, United States). The block dimensions were 39 cm × 19 cm × 15 cm, with a mass of 14 kg each. A subset of polyps from each colony were preserved immediately in 99.5% ethanol for subsequent molecular analyses of symbiont communities. The blocks were then returned to the depth of origin to acclimate. Colonies were not subfragmented, so the colonies on each block from each depth of origin were distinct (there was no pseudoreplication of clonal fragments) and likely represented unique genotypes, though this was not confirmed.

After 3 days of acclimation, the blocks were transplanted among depths, following a standard reciprocal design with four treatments (Figure 1). One intertidal origin block remained at 2 m as a control (“shallow-to-shallow” or SS; *n* = 20 colonies), while another was moved to 26 m (“shallow-to-deep” or SD; *n* = 20); one subtidal origin block remained at 26 m as a control (“deep to deep” or DD; *n* = 20), while another was moved to 2 m (“deep-to-shallow” or DS; *n* = 20). Blocks remained at each depth for approximately 2 months. Individual polyps from each living colony were sampled on SCUBA at the start of the experiment (all transplants), at 1 week (DS transplant only), and at 4 weeks (all transplants) and fixed in 99.5% ethanol. All colonies were observed at these time points as well as at 8 weeks to record their health status as alive (apparently healthy), dead (>95% tissue loss), or missing (cases where the entire substrate was absent).

### DNA Extraction and Phylogenetic Analyses

All samples were initially screened to identify the numerically dominant symbiont species *via* direct Sanger sequencing. Total genomic DNA was extracted using a DNeasy Blood & Tissue Kit (QIAGEN Inc., Tokyo, Japan). Symbiodiniaceae DNA was amplified with a HotStarTaq Plus Master Mix Kit (QIAGEN, Inc., Tokyo, Japan) targeting the nuclear internal transcribed spacer 2 (ITS2) region with the primers ITSintfor2 (5'-GAATTGCAGAACTCCGTG-3') and ITS-reverse (5'-GGGATCCATATGCTTAAGTTCAGCGGGT-3'; LaJeunesse and Trench, 2000), as this marker can clearly resolve *Symbiodinium* spp. from *Cladocopium* spp., and weakly resolve C1z-shallow and C1z-deep (Kamezaki et al., 2013). For additional resolution, the noncoding region of the chloroplast psbA minicircle (psbA^ncr^) was amplified using the primers 7.4-Forw (5'-GCA TGAAAGAAA TGCACACAACTTCCC-3') and 7.8-Rev (5'-GGTTCTCTTATTCCATCAATATCTACTG-3'; Moore et al., 2003).

Amplifications were performed in 20 μl reaction volumes containing 10 μl of Master Mix, 7 μl of ultrapure water, 1 μl of each forward and reverse primer (final concentration: 1 μM), and 1 μl of template DNA. Thermocycler conditions for ITS2 were as follows: 5 min at 95°C; 35 cycles of 45 s at 94°C, 45 s at 54°C, and 30 s at 72°C; and a final extension of 10 min at 72°C. Thermocycler conditions for psbA^ncr^ were as follows: 5 min at 95°C; 40 cycles of 10 s at 94°C, 30 s at 55°C, and 2 min at 72°C; and a final extension of 10 min at 72°C. Products were cleaned by the addition of 2.55 μl of ultra-pure water, 0.15 μl of Exonuclease I, and 0.3 μl of Shrimp Alkaline Phosphatase (TAKARA Co., Ltd., Shiga). Thermocycler conditions were as follows: 20 min at 37°C, followed by 30 min at 83°C. Cleaned products were shipped frozen to FASMAC (Kanagawa, Japan) for sequencing.

The ITS2 and psbA^ncr^ nucleotide sequences were aligned within Geneious v9.1.3 (Biomatters, New Zealand) and inspected manually. Sequences were aligned to references in GenBank (National Center for Biotechnology Information) to confirm identity. All ITS2 sequences matched previous references. Due to the short length of some of the psbA^ncr^ sequences, they could not be deposited in GenBank; instead, alignments are provided in the Supplementary Figure S2. Distance, parsimony, and maximum likelihood bootstrap consensus cladograms were generated in PAUP* v4a169 (Swofford, 2001), using the optimum inferred nucleotide evolution model (Jukes-Cantor for both *Symbiodinium* and *Cladocopium* alignments) and 1,000 bootstraps to calculate node support. Genetic distances among representative psbA^ncr^ sequences for C1z-shallow and C1z-deep were calculated with MEGA v6.0 (Tamura et al., 2013) and compared to divergence among other closely-related *Cladocopium* species (Thornhill et al., 2014).

### Relative qPCR

Three species-specific primer sets targeting the Symbiodiniaceae psbA^ncr^ region were designed for A1z-shallow, C1z-shallow, and C1z-deep (Table 1). Appropriate, unique primers for each sequence were identified using the online tool Primique (Fredslund and Lange, 2007). Reactions were performed using SYBR Green chemistry on an ABI StepOne Plus Real-Time PCR System (Thermo Fisher Scientific, MA, United States). Reaction volumes consisted of 10 μl Fast SYBR Green Master Mix (Thermo Fisher Scientific), 7 μl purified water, 1 μl of each forward and reverse primer (final concentration: 0.5 μM), and 1 μl template DNA. Thermocycler conditions were as follows: 10 min at 95°C; 40 cycles of 3 s at 95°C followed by 30 s at 60°C; 15 s at 95°C; 1 min at 60°C; concluding with a melting curve (continuous ramping of +0.3°C per cycle up to 95°C for 15 s each). Each reaction was performed in duplicate, and for any given sample all three species assays were included on the same plate. Each plate also included no-template controls and four 10-fold serial dilutions of reference samples (10–0.01 ng/μl) for all three species. The dilutions were used to generate plate-specific calibration curves accounting for differences in reaction efficiencies among assays and runs. Reference samples were chosen based on initial symbiont identification using direct PCR and subsequent quantitative PCR (qPCR) confirmation that the target species represented >99% of the symbiont community in each reference. Because the goal was relative quantification rather than absolute quantification, it was not necessary to normalize to a reference gene.

**Table 1:** Species-specific qPCR primer sets developed to amplify the chloroplast psbA^ncr^ region of Symbiodiniaceae associated with *Z. sansibaricus* in Okinawa, Japan.

Species	Abbreviation	Forward primer	Reverse primer	Mean efficiency
*Symbiodinium* sp. A1z	A1z-shallow	5'-CCACGAGGGTGGAAATGAGCTG-3'	5'-AATGCGAAGTATTGCGCTGGAC-3'	109.4%
*Cladocopium* sp. C1z-intertidal	C1z-shallow	5'-ACCCATAATCTTGGCCTGCTTG-3'	5'-CTTTCTCCTGCGGGCTCCTG-3'	87.1%
*Cladocopium* sp. C1z-subtidal	C1z-deep	5'-GACCACAATTTAGGCCACATC-3'	5'-GCCCTCTAATGCACTTCGTG-3'	82.5%

Within the StepOne Plus software, cycle threshold (C_T_) values were converted to initial concentrations based on the species-specific calibration curves for the plate on which the sample was run. These values were directly compared to compute the relative proportions of different Symbiodiniaceae species in each sample and plotted within the R statistical environment. Although amplification efficiencies were comparable between the *Cladocopium* assays, the *Symbiodinium* assay could not be optimized to fall within a similar range (Table 1). The over-efficiency of this assay likely shifted C_T_ values such that it appeared there were more *Symbiodinium* cells and fewer *Cladocopium* cells when combinations of both genera were present in a sample. However, high consistency between the output of direct sequencing and qPCR methods indicate that despite the qPCR bias toward *Symbiodinium*, the assays captured which species were dominant, and any inaccuracies in the observed relative abundances did not influence the key findings (see Results and Discussion).

### Intracolony Sampling

Results from the reciprocal transplant time series seemed to suggest that in the “shallow-to-shallow” treatment, intertidal *Z. sansibaricus* shuffled their symbiont communities back and forth among A1z-shallow and C1z-shallow in less than a month. Such rapid shuffling under control conditions was unexpected. An alternative explanation was that *Z. sansibaricus* in the shallows featured spatial variation in the distribution of symbiont species across the colony surface. Under such a scenario, the strategy of collecting a few polyps at each time point for molecular analyses may have created an artificial pattern of symbiont shuffling, as regions of the colony with distinct symbiont communities may have been sampled at different times. To test for this possibility, four additional intertidal *Z. sansibaricus* colonies were sampled from Manza in late Spring 2017. Five sub-samples were taken per colony: one central polyp, and four polyps located 20 cm away and oriented in the four cardinal directions (north, south, east, and west). All sub-samples were analyzed *via* qPCR as described above to assess symbiont community composition.

## Results

### Environmental Parameters and Mortality

**Figure 3:**
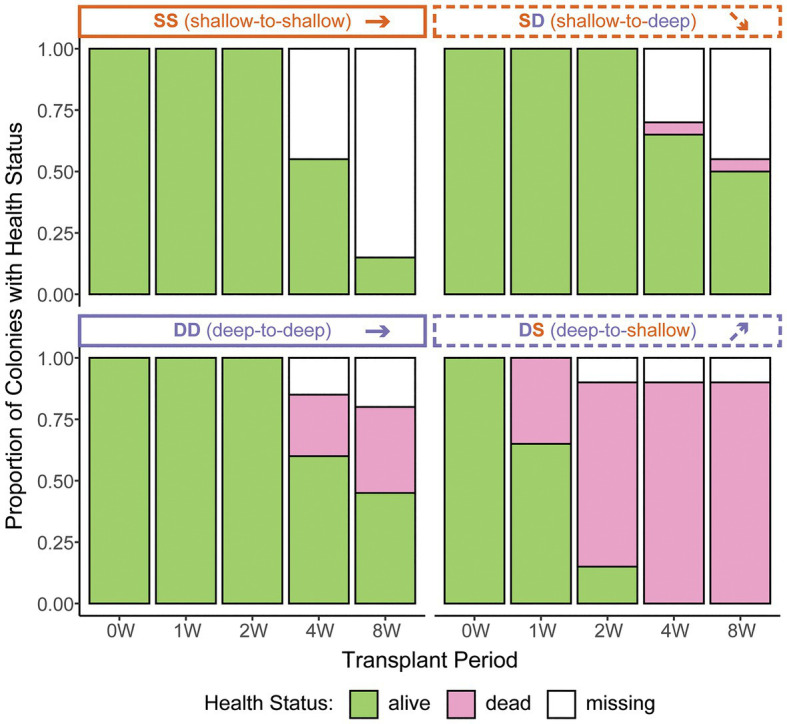
*Zoanthus sansibaricus* colony mortality over the course of the 2-month reciprocal transplant experiment at Manza in Summer 2013. “W” corresponds to the week of observation. Note the rapid mortality in the deep-to-shallow (DS) transplant, necessitating early sampling to characterize symbiont community composition. Anecdotally, all the living DS colonies were visually bleached at 1 and 2 W.

Based on 2 weeks of data from *in situ* loggers deployed in Summer 2015, Manza’s average daily maximum light intensity at 2 m was ~25-fold greater than at 26 m (25,142 lx ± 26,013 vs. 1,065 lx ± 493, respectively), and average daily maximum temperature was ~2.5°C warmer (31.1°C ± 2.8 vs. 28.5°C ± 0.2, respectively), with greater variability in the shallows (Figure 2). *Zoanthus sansibaricus* mortality varied by transplant, as did the incidence of missing colonies due to detachment of the substrate from the concrete block (Figure 3). All SS transplants that did not go missing survived, though this treatment also exhibited the greatest number of missing colonies (only three of 20 substrates remained attached after 8 weeks (8 W), but at 4 W there were still 11 living colonies). The SD transplants also showed low mortality (one of 11 colonies that did not go missing after 8 W expired). Missing colonies in the shallows likely resulted from large waves generated by summer typhoons (White et al., 2013). In the deep-to-deep (DD) transplant, mortality was greater (seven of 15 colonies that did not go missing after 8 W expired). Finally, the DS transplant was the most sensitive. After just 1 W in the shallows, seven of 20 colonies expired, and several of the 13 living colonies were completely bleached, while others were starting to pale (Fujiwara et al., personal observation). By 2 W, an additional eight colonies had expired, and two had gone missing, leaving only three living colonies. By 4 W, all remaining colonies had died. Given the rapid mortality observed in the DS transplants after 1 W, emergency sampling was carried out. Thus, symbiont communities in the DS transplants could only be compared at 0 W (pre-transplantation) and 1 W (non-bleached or pale colonies only). In the other treatments, it was possible to compare 0–4 W. Logistical issues prevented tissue sampling at 8 W. Due to mortality, missing colonies, and occasional DNA preservation failures, only the subset of colonies with complete data pairs for both pre‐ and post-transplantation were analyzed and presented (*n* = 9 per treatment).

### Confirming Symbiodiniaceae Species Identities

Direct sequencing of ITS2 revealed the numerically dominant Symbiodiniaceae species in each sample (69 of 72 cases, with three amplification failures). Across the entire experiment, only three unique ITS2 sequences were recovered. We aligned these sequences against previously published entries in the NCBI Genbank database to confirm they corresponded to the expected *Symbiodinium* and *Cladocopium* lineages. Consistent with previous characterizations of *Z. sansibaricus* symbiont communities at Manza (Kamezaki et al., 2013), the single *Symbiodinium* sequence was identical to that of ITS2 type A1z (accession JQ762357), which differs by one base pair from that of *Symbiodinium microadriaticum* (ITS2 type A1; accession AF333505). The two *Cladocopium* sequences were identical to that of ITS2 type C1z-shallow (accession JQ762324) and ITS2 type C1z-deep (accession JQ762330), which differ by 1–2 base pairs from that of *Cladocopium goreaui* (ITS2 type C1; accession AF333515). All A1z-shallow and C1z-shallow sequences were recovered from intertidal origin colonies, while all C1z-deep sequences were recovered from subtidal origin colonies.

**Figure 4:**
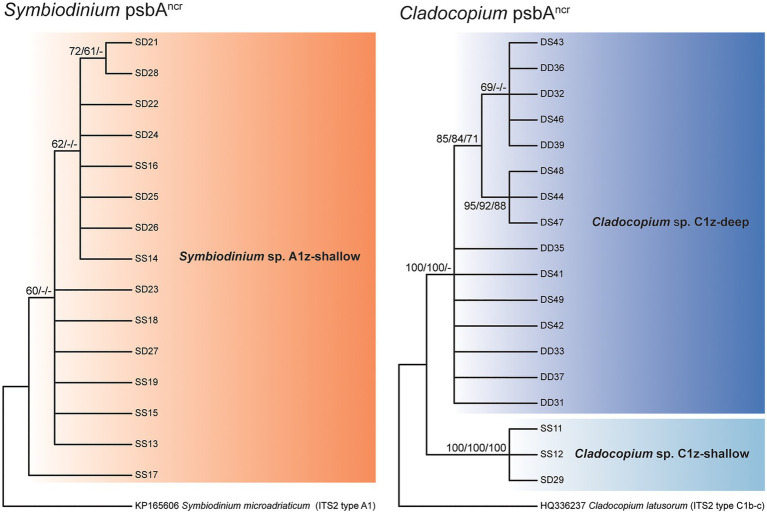
Consensus cladograms of the noncoding region of the chloroplast psbA minicircle (psbA^ncr^) sequences for *Symbiodinium* spp. (left) and *Cladocopium* spp. (right) based on distance methodology. Node values indicate bootstrap support (values below 60% excluded). Values correspond to different analyses in the following order: distance/parsimony/maximum likelihood. Dashes indicate where nodes were not supported by the given method.

Direct sequencing of the psbA^ncr^ also revealed the numerically dominant Symbiodiniaceae species in each sample. Where these sequences were successfully recovered, the results were in complete agreement with the ITS2 data (60 of 60 cases). Though more variable than ITS2, the *Symbiodinium* psbA^ncr^ sequences formed a single monophyly corresponding to A1z-shallow (no node support > 75%) and the *Cladocopium* psbA^ncr^ sequences split into two major monophylies corresponding to C1z-shallow and C1z-deep (node support = 100%; Figure 4). In a previous study, microsatellite data confirmed that sympatric populations of *Cladocopium* lineages corresponding to Caribbean ITS2 types C3, C7, and C7a were reproductively isolated and thus likely represent distinct species (Thornhill et al., 2014). The psbA^ncr^ sequences from these Caribbean *Cladocopium* species provided a frame of reference for inferring whether C1z-shallow and C1z-deep are divergent enough to constitute separate species. The genetic distance between C1z-shallow and C1z-deep (0.37) exceeded the average genetic distance among ITS2 types C3, C7, and C7a (0.17; maximum 0.23), so they are likely to represent distinct species, and we treat them as such in this study. The C1z-deep monophyly segregated into two subgroups (node support > 75%), but the genetic distance between these subgroups (0.01) fell below the average within-species distance among ITS2 types C3, C7, and C7a (0.05; minimum 0.02), so the subgroups are unlikely to represent distinct species, and we treat them here as one lineage.

### Changes in Symbiont Community Composition After Transplantation

Quantitative PCR of the psbA^ncr^ primers revealed not only the numerically dominant Symbiodiniaceae species, but also the abundance of background levels of the other focal species. These qPCR results were consistent with the psbA^ncr^ direct sequencing results in all but two instances (63 of 65 cases). For colony SD21 at 0 W, direct sequencing indicated that A1z-shallow dominated, while qPCR indicated that C1z-shallow dominated; for colony SD26 at 4 W, direct sequencing indicated that C1z-shallow dominated, while qPCR indicated that A1z-shallow dominated. Thus, even though the qPCR primer efficiencies were unequal across assays (Table 1), the bias toward *Symbiodinium* does not appear to have had a great effect in most samples, at least in terms of detecting the dominant species. The bias also reinforces that we did not detect A1z-shallow in nearly all deep-sourced colonies (see below), as any A1z-shallow signal would have been exaggerated.

**Figure 5:**
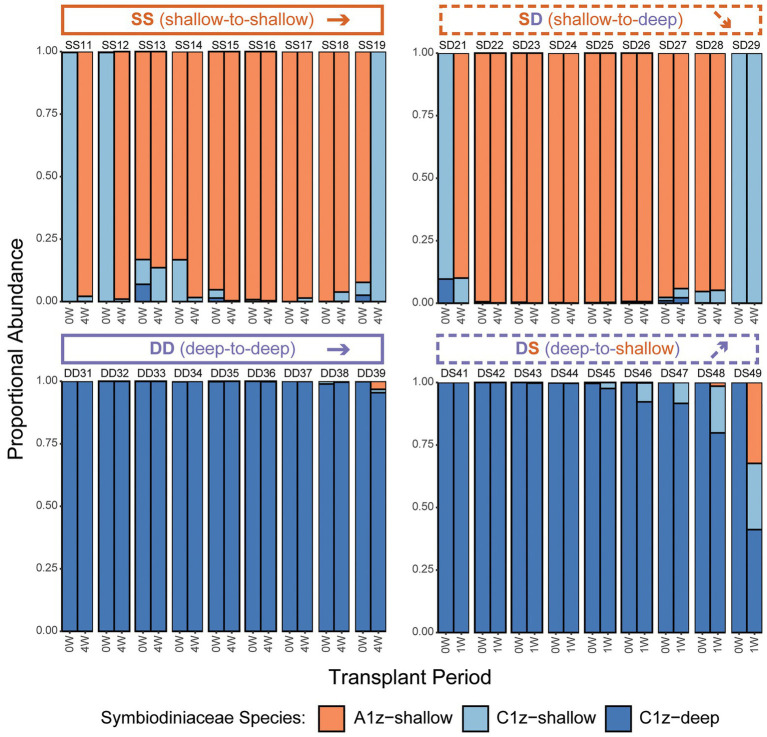
Changes in Symbiodiniaceae community composition for individual *Z. sansibaricus* colonies over the course of the reciprocal transplant experiment based on species-specific quantitative PCR (qPCR) during Summer 2013. “W” corresponds to the week of observation. Note that in the DS transplant, the final symbiont community characterization was made at 1 W instead of 4 W due to the onset of rapid mortality and visual bleaching among these colonies. Designations above barplots indicate transplant treatment (two letters) and colony ID (two numbers).

After transplantation, the first consideration was whether any colonies shuffled between intertidal Symbiodiniaceae species (A1z-shallow and/or C1z-shallow) and subtidal Symbiodiniaceae species (C1z-deep). Based on species-specific qPCR assays, there was no successful transplantation-induced shuffling of symbionts to favor species originating from different depths. All colonies of shallow origin (SS, *n* = 9 of 9; or SD, *n* = 9 of 9) began with a community composition consisting of >90% shallow symbionts prior to transplantation and ended with >90% shallow symbionts 4 W after transplantation (Figure 5). All but two colonies of subtidal origin (DD, *n* = 9 of 9; or DS, *n* = 7 of 9) began with >90% deep symbionts prior to transplantation and ended with >90% deep symbionts either 4 W (DD) or 1 W (DS) after transplantation (Figure 5). The two colonies with larger shifts were both in the DS treatment, which was experiencing rapid mortality after just 1 W. One colony (DS48) shuffled from ~100% C1z-deep to ~80:20% C1z-deep:C1z-shallow, while the other (DS49) shuffled from ~100% C1z-deep to 40:25:30% C1z-deep:C1z-shallow:A1z-shallow. Recall that the A1z-shallow signal in this colony may have been exaggerated by primer inefficiencies.

Because there were two intertidal Symbiodiniaceae species, the second consideration was whether there was any shuffling between these shallow symbiont species (A1z-shallow and C1z-shallow) within intertidal-origin colonies (SS or SD). Such transitions were present in both SS and SD transplants, but there was no predominant pattern. Some colonies began ~100% A1z-shallow and transitioned to ~100% C1z-shallow after 4 W (e.g., SS11), some showed the opposite (e.g., SS19), and others were intermediate between these extremes (Figure 5).

### Intracolony Spatial Variation in Symbiont Community Composition

**Figure 6:**
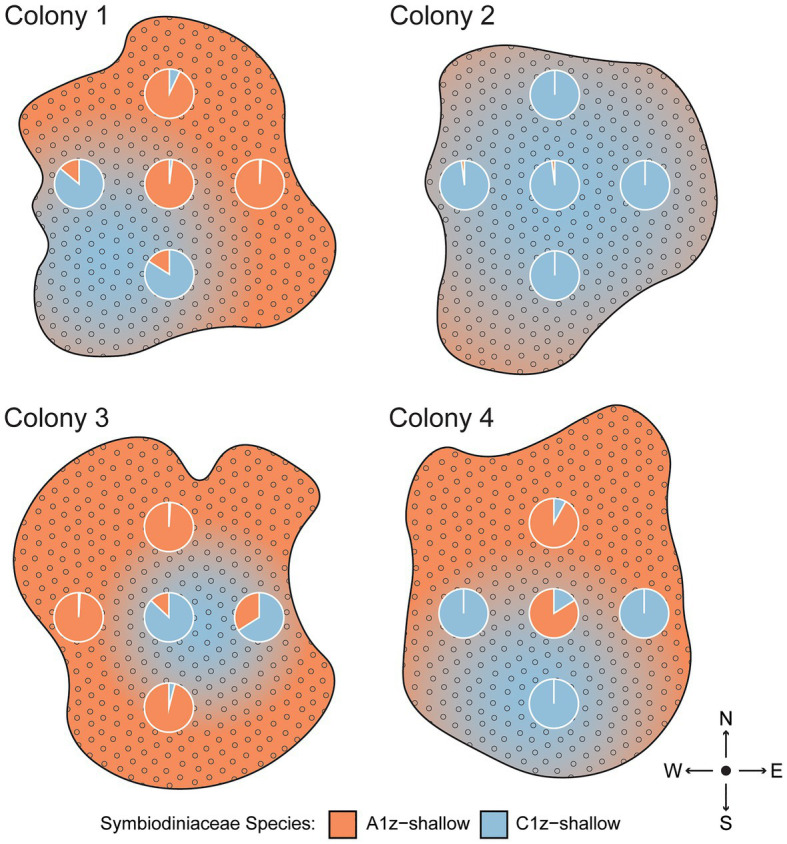
Spatial differences in Symbiodiniaceae community composition across the surfaces of four intertidal *Z. sansibaricus* colonies collected from Manza’s intertidal zone (2 m) in Spring 2017. Each colony was sampled at five points, represented by the pie charts of proportional abundance for each symbiont species. The gradients are subjective interpretations of symbiont distributions inferred from these points.

Because rapid shuffling among intertidal Symbiodiniaceae species within SS and SD transplants was unexpected, an alternative hypothesis of spatial variation in symbiont community composition was tested by subsampling four separate intertidal colonies five times each across the colony surface. Based on the same species-specific qPCR assays, it was confirmed that different regions of shallow colonies had different symbiont compositions (Figure 6). One colony (Colony 2) was uniformly dominated by C1z-shallow, while the remainder featured different mixtures of A1z-shallow and C1z-shallow at different points along the surface. The different species distributions appeared to be random with respect to cardinal direction.

## Discussion

Consistent with previous studies (Kamezaki et al., 2013; Noda et al., 2017), we confirmed that zoantharians in Okinawa can host several distinct symbiont species over small spatial scales (Figure 4). In the case of *Zoanthus sansibaricus*, not only were symbionts structured along a depth gradient representing distinct light and temperature regimes (Figure 2), we also detected spatial variation in symbiont distribution within individual intertidal colonies (Figure 6). In several cases, the surfaces of colonies were dominated by A1z-shallow in some regions and C1z-shallow in others. These patterns did not align with cardinal direction, indicating the distribution was unlikely to have been determined simply by orientation relative to the sun. During field collection, we noted that the shallow *Z. sansibaricus* colonies were often situated in small cracks in the intertidal zone reef substrate, with some portions protected in the shade and others more exposed to direct light (Fujiwara et al., personal observation), similar to other reefs in Japan (Reimer, 2008). This variation in irradiance at the scale of centimeters may explain the intra-colony symbiont community distribution, though this hypothesis will require further testing. Similar symbiont diversity within colonies has been documented in scleractinian corals, and has led to unique bleaching outcomes (Rowan et al., 1997; Brown et al., 2002; Kemp et al., 2014). The presence of spatial variation in shallow colonies is important to consider when interpreting apparent changes in symbiont community composition during the reciprocal transplant experiment.

Prior to transplantation, all shallow *Z. sansibaricus* colonies at Manza hosted A1z-shallow, C1z-shallow, or a combination of these two species of intertidal origin, while all deep colonies started with C1z-deep of subtidal origin (Figure 5). After transplantation, we found that nearly all colonies maintained stable associations: they were essentially “locked in” to the symbionts typical of their depth of origin, at least over the short time period examined in this study (Figure 5). The apparent shuffling between A1z-shallow and C1z-shallow in transplants of shallow colonies (the SS control and SD treatment) is most likely an artefact of intra-colony spatial variation in symbiont distribution (Figure 6), rather than rapid community changes. Although we cannot rule out rapid shuffling, it is a less parsimonious explanation than inadvertently sampling different regions of the colony at different time points, given our experimental design and the otherwise stable patterns of symbiont diversity. In addition to SD and DS transplants retaining their original symbionts, deep subtidal colonies visibly bleached and expired within 2 weeks of being brought to the shallow intertidal zone (Figure 3). These results match well with previous studies of reciprocally transplanted scleractinian corals (Baker et al., 2004; Frade et al., 2008; Sampayo et al., 2016) and experimentally manipulated sea anemones (Gabay et al., 2019; but see Herrera et al., 2020), and can be explained at least in part by specificity owing to symbiont niche-specialization.

As is true in other cnidarian-algal systems (Iglesias-Prieto et al., 2004; Frade et al., 2008), we can assume that the shallow Symbiodiniaceae at Manza are photo-adapted to the high-light, variable environment characteristic of the intertidal zone, while deep Symbiodiniaceae are photo-adapted to the low-light, constant environment of the subtidal zone. While shallow Symbiodiniaceae may still thrive at depth, especially over the short term, deep Symbiodiniaceae accustomed to lower light levels may face acute photoinhibition and possibly temperature stress in shallow environments, which can lead to dysbiosis, bleaching, and mortality (Baker et al., 2004; Frade et al., 2008; Sampayo et al., 2016; this study). One exceptional DS colony did appear to shuffle to a slight majority of shallow symbionts 1 week after being transplanted upward. However, there are reasons to think this may have also been an artefact. It is difficult to classify the extent to which zoantharians have visibly bleached (Parkinson et al., 2016), and although it was obvious that some DS transplants had started bleaching after just 1 week, the majority still looked healthy and were therefore sampled. The colony that shuffled between deep and shallow symbionts had relatively low DNA yield in the post-transplantation sample, indicating it may have already been bleaching despite appearing healthy. Therefore, it would be incorrect to interpret the outcome as successful shuffling – the symbiont community may have simply been in a state of flux due to stress-induced non-visible bleaching. Regardless of its bleaching status at 1 week, this colony ultimately died after 2 weeks, so while there may have been some variation among colonies in the capacity to change symbiont communities, such change was insufficient for survival. This suggests *Zoanthus*-Symbiodiniaceae associations are relatively stable, despite their ability to associate with more than one symbiont within a colony, and thus they appear unlikely to shuffle their symbiont communities to acclimate or adapt to rising sea surface temperatures caused by climate change. Further experimentation with more gradual manipulations over longer time scales will be required to confirm this prediction.

More generally, our results demonstrate that Symbiodiniaceae ecology is more important than phylogenetic relatedness in establishing a successful mutualism. This conclusion follows from the fact that intertidal *Z. sansibaricus* colonies that typically associate with the high-light adapted C1z-shallow will more readily accommodate the high-light adapted but evolutionarily divergent A1z-shallow than the low-light adapted but extremely closely related C1z-deep. The primacy of ecological compatibility is consistent with the distribution of other phylogenetically divergent Symbiodiniaceae within scleractinian coral colonies that can associate with multiple species simultaneously (e.g., Kemp et al., 2014). It is unclear to what extent the symbiosis discerns between C1z-shallow and C1z-deep *via* mechanisms like microbe-associated molecular patterns and pattern-recognition receptors (Davy et al., 2012; Parkinson et al., 2018). The exclusion of one *Cladocopium* species when both could be present might also stem from competitive interactions inherent to the symbionts (Gabay et al., 2019; McIlroy et al., 2019, 2020), which theory predicts to be of greater intensity among sister lineages than among more divergent lineages. Both host habitat and depth are major drivers of functional specialization in Symbiodiniaceae (Finney et al., 2010; Thornhill et al., 2014). This symbiont family has undergone multiple adaptive radiations over its evolutionary history, co-speciating with scleractinian corals and branching out to fill varied environmental niches and functional roles (LaJeunesse, 2005; Prada et al., 2014; Parkinson et al., 2015; LaJeunesse et al., 2018). The *Zoanthus*-Symbiodiniaceae association at Manza is unique in that the C1z lineage appears to have diverged along a depth gradient in the absence of genetic barriers in *Z. sansibaricus*. This highlights that Symbiodiniaceae typically exhibit more genetic structure than their hosts (Thornhill et al., 2017) and supports the contention that dinoflagellate endosymbionts may evolve more rapidly than cnidarians.

## Data Availability Statement

The datasets presented in this study can be found in online repositories. The names of the repository/repositories and accession number(s) can be found in the article/Supplementary Material.

## Author Contributions

YF, JR, and JP: conceptualization and formal analysis. YF, IK, JR, and JP: methodology, investigation, and writing–review and editing. JP: software. JR and JP: resources, supervision, project administration, and funding application. YF and JP: writing–original draft preparation and visualization. All authors contributed to the article and approved the submitted version.

### Conflict of Interest

YF was employed by the company Nakajima Suisan Co. Ltd., while the manuscript was being written, but after field experiments had been carried out.

The remaining authors declare that the research was conducted in the absence of any commercial or financial relationships that could be construed as a potential conflict of interest.

## Supplementary Material

Supplementary Figure S1 Raw data and R code.

Supplementary Figure S2 *Symbiodinium* and *Cladocopium* psbA^ncr^ sequence alignments.
